# Magnetic ressonance imaging in the diagnosis of Creutzfeldt-Jakob disease: Report of two cases

**DOI:** 10.1590/1980-57642015DN94000424

**Published:** 2015

**Authors:** Alan Peres Valente, Paula da Cunha Pinho, Leandro Tavares Lucato

**Affiliations:** 1 Setor de Neurorradiologia Diagnóstica. Instituto de Radiologia do Hospital das Clínicas da Faculdade de Medicina da USP.

**Keywords:** Creutzfeldt-Jakob disease, prionic disease, rapidly progressive dementia, MRI, diffusion, DWI, basal ganglia, cortex, Creutzfeldt-Jakob, doença priônica, demência rapidament progressiva, ressonância magnética, difusão, gânglios da base, córtex

## Abstract

Creutzfeldt-Jacob disease (CJD) is a rare condition caused by a pathogenic prion protein that evolves with rapidly progressive dementia and death. The clinical presentation may sometimes be misleading. Magnetic Resonance Imaging (MRI) aids diagnosis with patterns that can guide or confirm clinical hypotheses. Two cases of rapidly progressive dementia with ataxia, myoclonus and restricted diffusion on MRI in cortical/basal ganglia are presented to draw attention to CJD.

## Abstract (pt)

Doença de Creutzfeldt-Jacob (CJD) é uma rara doença relacionada a uma proteína priônica patogênica que evolui com demência rapidamente progressiva e morte. Por vezes, a apresentação clínica é inespecífica e desafiadora. A ressonância magnética contribui para o diagnóstico com padrões de imagem que podem orientar ou confirmar as hipóteses diagnósticas baseadas na clínica. Serão apresentados dois casos de pacientes com a forma esporádica da doença.

## INTRODUCTION

Prion diseases (formerly known as spongiform encephalopathies) were first described in the beginning of last century. In 1966, a "transmissible in a virus-like manner" hypothesis conferred this group of diseases with the unique characteristic of being both infectious and inherited, with long incubation periods.^1,2^ Creutzfeldt-Jakob disease (CJD), kuru, variant Creutzfeldt-Jakob disease (vCJD), Gerstmann-Sträussler-Scheinker syndrome (GSS), and fatal familial insomnia (FFI) are the five human prion diseases currently recognized.

Sporadic form Creutzfeldt-Jacob disease (sCJD), a condition with rapidly progressive dementia and a fatal outcome, accounts for the majority of these very rare entities. The iatrogenic (iCJD) form (related to dural implants / cornea transplants) and the familial form (fCJD) both have, in common with sCJD, the pathological human prion protein (PrPsc) related to the prion protein gene.^1^ Variant form (vCJD) is caused by the transmission of the bovine spongiform encephalopathy agent to humans and is considered a distinct entity.

Progressive mental deterioration and myoclonus are the most important and typical symptoms for diagnosis.^3^ Cerebellar and oculomotor symptoms are common, and sometimes signs of pyramidal/extrapyramidal dysfunction can also be present. Electroencephalogram patterns (EEG), especially periodic sharp wave complexes, and detection of 14-3-3 protein in cerebrospinal fluid can be adjunctive in the diagnosis of sCJD, with good sensitivity and specificity. Magnetic resonance imaging (MRI), particularly the technique using diffusion-weighted images (DWI),also plays an important diagnostic role.^3,4^

In the present study, we present two sCJD cases with classic clinical presentations and imaging findings.

## CASE 1

A 51-year-old female patient with no previous medical history presented at the emergency department with gait disturbance and rapidly progressive cognitive decline, which developed over 4 months. The patient was disoriented with psychomotor slowing. She could not assume orthostasis or walk without assistance although the strength of her lower limbs was preserved. Myoclonus were evident and she had cerebellar symptoms (ataxia and dysdiadochokinesia). Sensitivity was low in the feet and fingertips.

MRI disclosed restricted diffusion in the basal ganglia (caudate and putamen), medial portion of the posterior thalami, frontal lobe cortex, anterior portion of the temporal lobes and insula in a symmetrical pattern. Posterior fossa structures were preserved. The majority of these areas had corresponding high signal on T2-weighted and FLAIR images.

Laboratory investigation was extensive, and entirely negative. CSF exam disclosed no remarkable findings.

Based on MRI pattern and clinical scenario, a diagnosis of sCJD was reached.

## CASE 2

A 61-year-old male patient was admitted to our service with a 5-month history of difficulty walking, writing and speaking, together with muscular spasms and limitation in daily activities. EEG showed disorganized slow waves while 14-3-3 protein was present in CSF samples. No other relevant laboratory findings were present.

MRI showed symmetrical restricted diffusion in the basal ganglia (caudate and putamen), medial and anterior portion of the cortex of frontal lobes and insula. Thalami were also involved.

## DISCUSSION

MRI plays an increasingly important role in the diagnosis of sCJD and, in combination with clinical, EEG and 14-3-3 protein, is part of the WHO criteria for probable and possible CJD.^5^ Definitive diagnosis is reached only by histopathological study. Sensitivity and specificity for typical MRI findings lie in the 83-92% and 87-95% ranges, respectively.^6,8^

Although standardized protocol exists, T2-weighted and FLAIR images are fundamental, together with diffusion-weighted image (DWI) sequences, for greater sensitivity and specificity. These sequences are now usually part of routine protocols.

Hyperintensity on T2-weighted and FLAIR images involving cortex and basal ganglia (especially the head of the caudate nucleus and putamen) associated with progressive brain atrophy are typical, but sometimes the hyperintensity is very subtle or not detectable in early phases. Other structures may also exhibit signal abnormalities such as the globus pallidus, thalamus, white matter and cerebellar cortex.

DWI is a fast sequence and widely used in MRI protocols. It has a greater sensitivity (range 80-100%)^4,6,7^ than T2/FLAIR images, especially for cortical involvement in early stages of the disease.^4,6,8^ DWI sequences may precede EEG and laboratory tests, when dealing with early diagnosis of sCJD.

A low apparent diffusion coefficient (ADC) may be associated with DWI hyperintensity.^9^ Some authors have sought to correlate low ADC values, particularly in the thalamus, with spongiform changes and accumulation of the pathologic form of PrP, but there is some controversy on this matter.^10,11^ ADC values vary dynamically with disease progression, and can be low even when normal signal intensities are found on other sequences such as FLAIR and T2-weighted. This finding might be correlated to a rapid change in composition of diseased tissue. High ADC values are more commonly associated with atrophy and gliosis.

Regarding regions of involvement, cortex is the most prevalently affected in the literature.^4^ Some distinct presentations have been defined based on clinical neurological findings and pattern of MRI involvement, such as subtypes with mainly cerebellar (Oppenheimer-Brownell variant) and occipital/visual cortex (Heidenhain variant) changes. Cortical hyperintensity is described in the literature as the ribboning sign, and can occur in a symmetrical or asymmetrical fashion.^12^

Basal ganglia (caudate and putamen) are the second most prevalent region of signal disturbance on MRI, although some authors claim this is the most prevalent. Association with cortical changes are suggestive of CJD. The globus pallidus is seldom involved, being more typically affected in late disease. Abnormal periaqueductal gray matter and posterior deep white matter changes may be present in vCJD.^13^

Thalamic involvement, initially described as a characteristic feature of vCJD, can also be found in sCJD, and histopathological findings of thalamic lesions in post mortem specimens is very common.^9^ Nonspecific thalamic hyperintensity appears to be more frequent in sCJD (present in around 13% of cases).^4^ On the other hand, pulvinar hyperintensity, especially when more evident than in other structures, appears to be more specific for vCJD (sometimes characterizing the "hockey stick sign"), and is part of WHO criteria for this variant. The pulvinar sign can also be found in sCJD and is therefore not exclusive to vCJD.^9,12-14^

Molecular classification based on polymorphism at codon 129 of the prion protein gene has been proposed by some authors,^15^ claiming good correlation with clinical presentation and MRI patterns.^16^

Atypical presentations have been described, differing to classical cortical and basal ganglia DWI restriction.

To sum up, MRI is vital for the diagnosis of sCJD, helping to differentiate it from several other conditions and dementias. Images typically show abnormal signal on DWI sequences in the cortex, putamen and head of the caudate nucleus; but unusual patterns can also occur. Sensitivity and specificity for typical MRI findings lie in the 83-92% and 87-95% ranges, respectively.^6,8^ The primary objective of this article was to explore the classical MRI findings of sCJD.

In conclusion, characteristic MRI findings, especially using diffusion weighted images, play an important role in the diagnosis of sCJD.

## Figures and Tables

**Figure 1:**
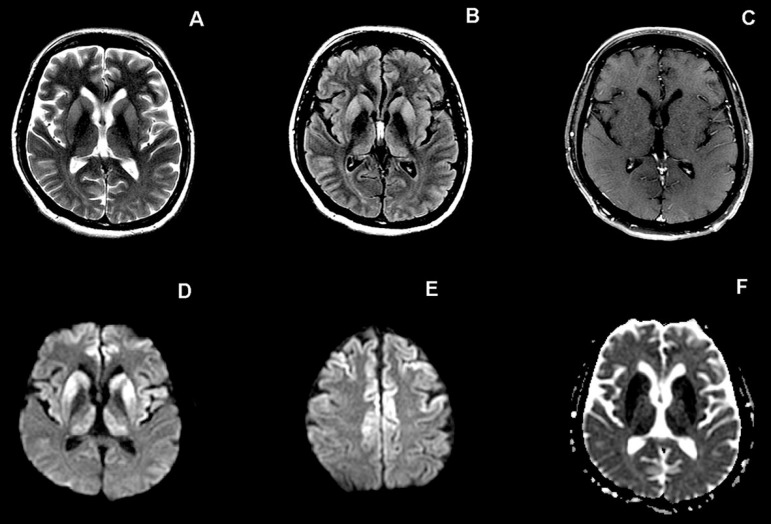
Case 1. Axial T2-weighted (A) and FLAIR (B) images: hyperintensity in basal ganglia, with less evident hyperintensity in cortex. Axial post-contrast T1-weighted image (C) shows no enhancement. Axial DWI (D, E): hyperintensity more evident than on T2/FLAIR images, involving the caudate nuclei bilaterally, putamen, medial portion of the thalami and both frontal and insular cortices. DWI-hyperintensity in basal ganglia and thalami have corresponding restricted diffusion on the ADC map (F).

**Figure 2:**
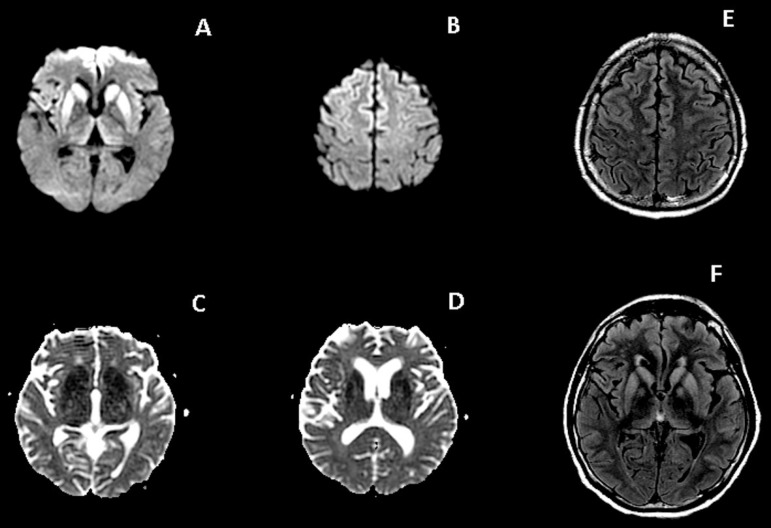
Case 1. Case 2: Axial DWI (A and B) shows hyperintensity in basal ganglia, thalami, insular and frontal cortex, with corresponding restricted diffusion in ADC maps (C and D). There is also high signal involving these structures on axial FLAIR images (E and F). The DWI sequence is more sensitive for depicting MRI changes related to CJD.
